# Evaluating the precision of *EBF1* SNP x stress interaction association: sex, race, and age differences in a big harmonized data set of 28,026 participants

**DOI:** 10.1038/s41398-020-01028-5

**Published:** 2020-10-20

**Authors:** Abanish Singh, Michael A. Babyak, Mario Sims, Solomon K. Musani, Beverly H. Brummett, Rong Jiang, William E. Kraus, Svati H. Shah, Ilene C. Siegler, Elizabeth R. Hauser, Redford B. Williams

**Affiliations:** 1 Behavioral Medicine Research Center, Duke University School of Medicine, Durham, NC USA; 2 Department of Psychiatry and Behavioral Sciences, Duke University School of Medicine, Durham, NC USA; 3 Department of Medicine of the University of Mississippi Medical Center, Jackson, MS USA; 4 Duke Molecular Physiology Institute, Duke University School of Medicine, Durham, NC USA; 5 Department of Medicine, Duke University School of Medicine, Durham, NC USA; 6 Department of Biostatistics and Bioinformatics, Duke University School of Medicine, Durham, NC USA

**Keywords:** Personalized medicine, Clinical genetics

## Abstract

In prior work, we identified a novel gene-by-stress association of *EBF1*’s common variation (SNP rs4704963) with obesity (i.e., hip, waist) in Whites, which was further strengthened through multiple replications using our synthetic stress measure. We now extend this prior work in a precision medicine framework to find the risk group using harmonized data from 28,026 participants by evaluating the following: (a) *EBF1* SNPxSTRESS interaction in Blacks; (b) 3-way interaction of *EBF1* SNPxSTRESS with sex, race, and age; and (c) a race and sex-specific path linking *EBF1* and stress to obesity to fasting glucose to the development of cardiometabolic disease risk. Our findings provided additional confirmation that genetic variation in *EBF1* may contribute to stress-induced human obesity, including in Blacks (*P* = 0.022) that mainly resulted from race-specific stress due to “racism/discrimination” (*P* = 0.036) and “not meeting basic needs” (*P* = 0.053). The *EBF1* gene-by-stress interaction differed significantly (*P* = 1.01e−03) depending on the sex of participants in Whites. Race and age also showed tentative associations (*P*s = 0.103, 0.093, respectively) with this interaction. There was a significant and substantially larger path linking *EBF1* and stress to obesity to fasting glucose to type 2 diabetes for the *EBF1* minor allele group (coefficient = 0.28, P = 0.009, 95% CI = 0.07-0.49) compared with the same path for the *EBF1* major allele homozygotes in White females and also a similar pattern of the path in Black females. Underscoring the race-specific key life-stress indicators (e.g., racism/discrimination) and also the utility of our synthetic stress, we identified the potential risk group of *EBF1* and stress-induced human obesity and cardiometabolic disease.

## Introduction

Understanding the precise role of genetic, demographic, and environmental variations on the expression of complex biological mechanisms contributing to the development and course of major medical disorders is critical for next-generation medicine, often referred to as precision medicine^1,2^. Its framework defines the human diseases at greater resolution by focusing on a particular target risk group or subpopulation^3^ based on several factors, such as individual’s genetic and molecular makeup; complex physiological aspects of race, sex, and age; lifestyle and environmental factors; and their interactions. Although the progress in precision medicine research has been slow to develop^4^, there are several examples of success in this emerging field^5^, which support the contention that precision medicine approaches have the potential to help with the safety and effective delivery of health solutions to the target risk groups^6^. Two among the many challenges in achieving clinical utility of the precision medicine framework are accomplishing data pooling (i.e., data harmonization) in order to reconcile the evidence from multiple ongoing investigations^7^ and developing robust estimates of the interactions among an individual’s genetic makeup and complex physiological aspects of race, sex, and age. Our past and current work have focused on both of these challenges. In prior work, we have accomplished the integration of heterogeneous data sets collected from multiple sources, harmonizing inconsistencies among measurement protocols, units, and coding^8^. In the current work we focus on developing generalizable robust estimates within specific strata of the interactions using this large harmonized data set.

Our central working hypothesis holds that chronic psychosocial stress modifies the association between genetic variants and cardiovascular disease (CVD) risk at key phenotypic nodes (e.g., obesity) along the disease pathways^9^. In our previous study^10^, we identified a novel CVD-risk gene *EBF1* using the Multi-Ethnic Study of Atherosclerosis (MESA) cohort White samples, wherein the presence of chronic psychosocial stress a common variation (SNP rs4704963) influenced individual differences in central obesity—hip circumference as primary phenotype. We further identified a statistically significant path linking stress and *EBF1* genotype to obesity to fasting glucose to CVD risk^10^, confirming our central hypothesis^9^. We also observed similar gene-by-stress associations with waist circumference as an obesity trait^10^. Our efforts to replicate this finding^10^ in other data sets were challenged by the absence of an explicit measure of psychosocial stress. In response, we created a synthetic measure of psychosocial stress in such data sets^11^. Our synthetic stress algorithm is based on the use of proxy items in the domains of the formal, self-rated measure of chronic psychosocial stress from the MESA, based on information about financial, marital, work, own health, and health of a spouse or someone close^12^. We achieved replication of the *EBF1* SNPxSTRESS association with obesity, however, also only in White samples. One of the possible reasons for not originally finding (at genome-wide level) or achieving replication (at level *P* ≤ 0.05) in Black samples may be the use of MESA-like five components of chronic psychosocial stress measure, which may not be sufficient to capture the stress in everyday life among the Black participants. This demographic subgroup-specific (i.e., only in Whites) gene-by-stress association of *EBF1* made it an ideal candidate for the precision medicine analytic framework. In the current study, we used the MESA data set for the initial observations on race and sex-stratified analysis in order to formulate several testable hypotheses (as described in the “Materials and method” section) that were related to the variability we observed in the results of fitting a gene-by-stress interaction. Our hypotheses were focused on the following problems: (a) failure to observe a significant *EBF1* gene-by-stress association in Black samples, (b) observing a significant association only in a specific subgroup of samples (i.e., White females), and (c) extending our understanding of the clinical implications of race and sex-specific gene-by-stress associations linking to a path from *EBF1* and stress to obesity to fasting blood glucose to the development of cardiometabolic disease risk (e.g., type 2 diabetes mellitus). We used harmonized data sets from 28,026 participants derived from ten studies^8^, including the Jackson Heart Study^13^, for testing these hypotheses. In this report, we present the utility of our synthetic stress in harmonized data sets; additional efforts to show, including in Black samples, that common variation in *EBF1* may contribute to inter-individual differences in human obesity in the presence of stress; a systematic evaluation of sex, race, and age interactions with *EBF1* gene-by-stress association to identify the precise risk group; and also the evaluation of its clinical implication, i.e., a path linking *EBF1* and stress to obesity to fasting blood glucose to the development type 2 diabetes mellitus in the risk group.

## Materials and methods

### Study data sources

We used a harmonized data set that was derived from ten studies^8^, including six large cohort public-access studies and four smaller Duke studies. The public-access data sets were from the Jackson Heart Study (JHS)^13^; The Women’s Health Initiative (WHI) Study^14^; The Coronary Artery Risk Development in Young Adults Study (CARDIA)^15^; Atherosclerosis Risk in Communities Study (ARIC)^16^; Framingham Offspring Cohort^17^; and Multi-Ethnic Study of Atherosclerosis (MESA)^18^. These public-access data sets were obtained from the dbGaP/database of Genotypes and Phenotypes/National Center for Biotechnology Information, National Library of Medicine (NCBI/NLM)/ https://www.ncbi.nlm.nih.gov/gap^19^ through authorized/controlled data access under the standard user agreement. The Duke data sets were the Duke Family Heart Study (DFHS)^20^; Duke Caregiver Study (DCS)^21^; and two cohorts for Studies of a Targeted Risk Reduction Intervention through Defined Exercise (STRRIDE), i.e., STRRIDE—Aerobic Training/Resistance Training (AT/RT)^22^, and STRRIDE pre-diabetes (PD)^23^ studies. These Duke data sets were obtained from the studies conducted at Duke University Medical Center (DUMC). The accession of these data sets was approved by the Duke Institutional Review Board (IRB). A more detailed description of these study populations is provided in the Supplementary Materials.

### Data harmonization

The harmonization of data from the above-listed study cohorts of varying size and demography is described elsewhere in Singh et al.^8^, where we harmonized data sets for a measure of chronic psychosocial stress, candidate SNPs (e.g., *EBF1* rs4704963), and CVD-risk variables, including adiposity and hyperglycemia. Our focus for the current work was mainly on non-related samples of White and Black ancestries, thus, we used 28,026 White and Black participants (Table 1).

**Table 1:** Race and sex-stratified summary of study variables from harmonized data set comprising 28,026 non-related samples that were derived from ten studies.

Variable	White, *N* = 15,027				Black, *N* = 12,999				All, *N* = 28,026			
	Male, *N* = 7056		Female, *N* = 7971		Male, *N* = 2703		Female, *N* = 10,296		Male, *N* = 9759		Female, *N* = 18,267	
	Mean	SD	Mean	SD	Mean	SD	Mean	SD	Mean	SD	Mean	SD
Age (years)	52.52	12.43	51.89	12.21	50.69	14.64	56.74	11.5	52.01	13.1	54.62	12.06
Waist circum. (cm)	98.17	11.75	90.46	16.16	96.87	15.08	93.38	15.56	97.81	12.77	92.11	15.89
Hip circum. (cm)	102.87	7.87	104.07	11.34	103.54	10.2	110.66	12.83	103.03	8.5	107.69	12.62
Fasting glucose (mmol/L)	5.67	1.56	5.4	1.52	6.02	2.43	6.04	2.66	5.76	1.82	5.61	2.00
Synthetic stress scale, *z*-scores	−0.23	0.86	−0.08	0.94	0.19	1.06	0.18	1.07	-0.11	0.94	0.07	1.02
*Type 2 diabetes mellitus*												
0	90.64%		92.72%		83.16%		85.75%		88.63%		88.73%	
1	9.36%		7.28%		16.84%		14.25%		11.37%		11.27%	
rs4704963, MAF	0.071		0.073		0.016		0.02		0.055		0.043	
*Study-wise sample % in the harmonized data set in each stratum*												
ARIC	63.51%		63.05%		40.36%		17.59%		57.10%		37.43%	
CARDIA	9.64%		9.45%		14.24%		5.24%		10.91%		7.07%	
FRAMINGHAM	6.28%		6.02%		0%		0%		4.54%		2.63%	
JHS	0%		0%		16.39%		5.83%		4.54%		3.28%	
MESA	16.41%		15.82%		26.30%		7.69%		19.15%		11.24%	
WHI	0%		0%		0%		61.62%		0%		34.73%	
DCS	0.79%		1.91%		0.52%		0.62%		0.72%		1.18%	
DFHS	1.52%		1.69%		1.37%		0.87%		1.48%		1.23%	
STRRIDE-AT/RT	0.95%		0.98%		0.41%		0.28%		0.80%		0.59%	
STRRIDE-PD	0.91%		1.08%		0.41%		0.26%		0.77%		0.62%	

### Study variables

#### Genetic variant

For the proposed analysis, we used harmonized *EBF1* genetic variation data, i.e., SNP rs4704963 if available, or SNP rs17056278 (LD *R*^2^ = 1 with rs4704963)^8^. The SNP genotyping was done in dbGaP SHARe data sets MESA, CARDIA, WHI, ARIC, and JHS using the Affymetrix Genome-Wide Human SNP Array 6.0 and in the Framingham Cohort using the Affymetrix Mapping250K (Nsp and Sty) Arrays and Mapping50K (Hind240 and Xba240) Arrays. The SNP genotyping was done in the Duke data sets DFHS and DCS using ABI 7900 Taqman system (Applied Biosystems) platform and in STRRIDE using Taqman (Life Technologies) and the QuantiFast Multiplex PCR + ROX kit (Qiagen) platforms. The population ancestry principal components were created for the studies that included SNP array data sets. The SNP genotypes were subjected to standard quality control metrics^10^. The *EBF1* genotypes were available for all samples of the harmonized data set that were included in this study (Table 1).

#### Synthetic stress measure

Out of the ten harmonized studies, only two studies, the MESA and JHS, used a self-rated stress measure. The MESA stress measure was based on the following five domains: financial strains, relationship or marital problems, difficulties with job or ability to work, serious health problems of a spouse or someone close, and one’s own serious health problems^12^. However, the JHS stress summary measure was based on eight components, i.e., stress due to job, relationships, neighborhood, care-giving, legal problems, medical problems, racism/discrimination, and (not) meeting basic needs^24^. The three additional components in JHS were stress due to neighborhood, legal problems, and racism/discrimination. In addition, the financial strains component was evaluated differently, i.e., by using a more specific question on (not) meeting basic needs. For the remaining studies that did not have a self-rated stress measure, we constructed a synthetic stress measure as reported in our data harmonization efforts^8,11^ employing our algorithm that uses proxy indicators of the domains used in the MESA chronic burden measure^12^. Briefly, our algorithm^11^ searched for proxy indicators of each stress domain, scored each proxy item as 1 = stressful, 0 = not stressful. The item scores were then summed to obtain a single score, which varied in range across the studies due to the non-availability of all items in each study. We transformed the scores to z-scores (mean of zero and a standard deviation of one) within each study in order to harmonize the differently scaled measures. The list of proxy items for each study is provided in Supplementary Materials. More details of the construction of synthetic stress are provided elsewhere in our data harmonization efforts^8,11^.

In addition, we created MESA-alike and MESA-unlike partial stress summary scores in the JHS using its available individual stress components: For MESA-alike score, we used a total four out of the eight self-rated stress components, i.e., job, relationship, caring for, and medical problem; and for MESA-unlike score, we used four components that were additional to MESA-alike components or was a differently evaluate an item, i.e., “racism/discrimination”, “living in neighborhood”, “legal problems”, and “not meeting basic needs”.

#### Primary phenotypes and independent variables

In order to evaluate the *EBF1* gene-by-stress interaction in consistency with our discovery analysis^10^, we used the obesity trait hip circumference as the primary phenotype for the initial phase of analysis (i.e., observation/ hypotheses building, as described below in the Analysis section) in MESA data set. Hip circumference was not present in JHS, therefore, we used waist circumference as the primary phenotype for the final phase of analysis (i.e., hypotheses testing) in the JHS and harmonized studies’ data sets. We also used fasting blood glucose and type 2 diabetes mellitus (DM) status for evaluating the structural equation path model linking *EBF1* SNP and stress to obesity to fasting glucose to type 2 diabetes mellitus, a cardiometabolic disease risk^10^. Other study variables that were used as key independent variables in the analyses were demographic variables (age, sex) and population ancestry principal components (if the data were available for analysis). Table 1 shows a summary of these study variables.

### Analysis

The analysis proceeded in three phases—observation, hypotheses building, and hypotheses testing to evaluate race, sex, and age differences in the *EBF1* gene-by-stress association in the context of a precision medicine framework. We used the MESA data set for the observation and hypotheses building phases and JHS and combined (harmonized) data sets derived from the ten studies for the hypotheses testing phase.

### Observation phase

#### Race and sex-stratified analysis in MESA

We performed race and sex-stratified linear regression on the primary phenotype hip circumference in the MESA data set for the *EBF1* SNP rs4704963 under the additive genetic model, consistent with an original discovery analysis^10^. The interaction model included age, SNP, STRESS, and SNPxSTRESS along with population ancestry correction. We also evaluated the SNP main-effect using a conventional SNP-only additive model (i.e., without STRESS and SNP×STRESS terms). The ordinal stress variable was treated as a linear variable. The gene-by-stress interaction was tested by the SNPxSTRESS product term in the model and interaction was considered significant at the threshold *P*-value ≤ 0.05 for the single SNP analysis. We also plotted the race and sex-stratified distribution of the mean of hip circumference in MESA data set against each ordinal value of stress for the two genotype groups of *EBF1* SNP, i.e., major allele homozygotes (TT) and minor allele heterozygotes and homozygotes (CT/CC).

### Hypotheses building phase

We formulated the following testable hypotheses based on the outcomes of the above described observational phase of analysis:

**(1a)** that a five-point stress measure is not adequate to capture stress information in Black populations and thus one or more additional stress component(s) (specifically, “racism/discrimination”) or an existing component but evaluated differently (especially, financial strains component evaluated using the question on “not meeting basic needs”) may contribute in *EBF1* gene-by-stress interaction. We used the JHS data set to test this hypothesis.

**(1b)** that excluding the above additional or differently evaluated items from the stress score (i.e., making the stress score equivalent to MESA-like self-rated stress measure) may not result in the significant *EBF1* gene-by-stress interaction in the JHS data set.

**(2a)** that the significant *EBF1* gene-by-sex interaction was observed only in females and thus we may observe a 3-way SNPxSTRESSxSEX interaction.

**(2b)** that this interaction was significant in White MESA samples and thus we may observe a 3-way SNPxSTRESSxRACE interaction.

**(2c)** that the significant *EBF1* gene-by-sex interaction was initially observed in a relatively old population and thus there may be a 3-way SNPxSTRESSxAGE interaction.

**(3)** that there will be a sex and race-related difference in the implication of obesity-related *EBF1* gene-by-stress interaction to other cardiometabolic risk factors and clinical outcomes, such as, fasting glucose, type 2 diabetes mellitus status—i.e., there may be sex and race-related differences in the significance of path from stress to obesity to fasting blood glucose to type 2 diabetes mellitus status.

### Hypotheses testing phase

**(1)** We tested the *EBF1* gene-by-stress interaction in the JHS data set (Black samples) using its eight components stress summary score. We also tested *EBF1* gene-by-stress interaction in JHS Black samples using the MESA-alike and MESA-unlike partial stress score. In addition, we tested the *EBF1* gene-by-stress interaction using the individual items that were included in MESA-unlike partial stress score in the JHS data set, i.e., “racism/discrimination”, “living in neighborhood”, “legal problems”, and “not meeting basic needs”. The full model was specified as:

WAIST CM = SNP + AGE + SEX + STRESS + SNPxSTRESS + ancestry PCAs.

We also performed a sex-stratified analysis using the model.

**(2)** *Mega-analysis*: Using the harmonized data set of combined samples composed from the ten studies^8^, we fit the 3-way interaction terms SNPxSTRESSxSEX, SNPxSTRESSxRACE, and SNPxSTRESSxAGE in separate regression models. Each model included all subordinate terms of the 3-way interaction term—for example, a model to test SNPxSTRESSxSEX also included SNPxSTRESS, STRESSxSEX, SNPxSEX, SNP, STRESS, and SEX terms. The multiple sourcing of data in combined samples in mega-analysis was taken into account using dummy study variables. For details on the use of dummy variables, see Singh et al.^8^. We checked for departure from normality for the primary outcome (dependent) variable and did not perform a transformation. The full models were specified as:

1: WAIST CM = SNP + STRESS + AGE + SEX + RACE + SNPxSTRESS + SNP × SEX + STRESS × SEX + SNP × STRESS × SEX + Study Dummy Variables.

2: WAIST CM = SNP + STRESS + AGE + SEX + RACE + SNP × STRESS + SNP × RACE + STRESS × RACE + SNP × STRESS × RACE + Study Dummy Variables.

3: WAIST CM = SNP + STRESS + AGE + SEX + RACE + SNP × STRESS + SNP × AGE + STRESS × AGE + SNP × STRESS × AGE + Study Dummy Variables.

We evaluated these interactions in each stratum, i.e., sex interaction in White, Black, and two races combined; race interaction in male, female, and two sexes-combined; and age interaction in White, Black, and two races combined samples. The visualization of 3-way interactions was performed using an *R* function based on the generalized Johnson–Neyman (J–N) technique^25^. This was followed by evaluating the association of the 2-way interaction term SNPxSTRESS and the model variables-adjusted partial correlation of SNP and stress separately with waist circumference in each stratum of the data set, i.e., for male, female, and two sexes-combined in White and Black samples.

**(3)** *Structural equation path analysis*: We modeled the path linking stress and *EBF1* genotypes to obesity (waist circumference) to fasting glucose to type 2 diabetes mellitus as a cardiometabolic clinical risk factor using structural equations path models^10^. We used generalized structural equation modeling (GSEM) as implemented in STATA 15.1 (StataCorp LLC.) to evaluate race, sex, and *EBF1* genotype-stratified possible causal paths from stress to waist circumference to fasting glucose to type 2 diabetes mellitus status (0/1). Path analysis uses a series of simultaneous equations to estimate possible mediating paths. The magnitude of a mediating effect is then calculated by taking the product of all path coefficients along a given proposed path. We modeled waist circumference and fasting glucose using linear regressions and dichotomous type 2 diabetes mellitus status using logistic regression, i.e., logit link in Bernoulli family as implemented in GSEM, and all analyses were adjusted for age. We implemented the multiple sourcing of data sets (i.e., combining data from ten studies) in the analyses by using study variable-based clustering of variance–covariance estimates and clustered robust standard errors. Our model was expressed as three simultaneous equations: (1) diabetes mellitus = b1*fasting glucose + b2*waist + b3*stress + b4*age; (2) fasting glucose=b5*waist + b6*stress + b7*age; and (3) waist =b8*stress + b9*age. In each equation, the term on the left is equivalent to the dependent variable in a regression-type model, while the terms on the right are the predictor variables. The coefficients b1 through b9 represent the regression slopes of each association. The three path model equations also imply three mediated effects from stress to type 2 DM: stress ⇒ waist ⇒ DM; stress ⇒ glucose ⇒ DM; and stress ⇒ waist ⇒ glucose ⇒ DM. We estimated simultaneous models for the TT and CT/CC groups in each category based on race- and sex-stratification. Further technical details of the structural equation modeling are shown in the Supplementary Materials.

## Results

### Observation phase

**Fig. 1:**
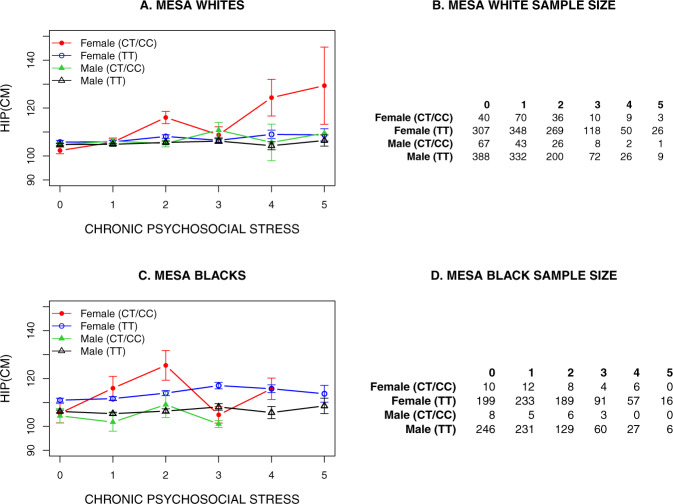
Direction of GxE association. The race and sex-stratified mean of hip circumference vs. chronic psychosocial stress for the two genotype groups of the *EBF1* SNP rs4704963, i.e., major allele homozygotes (TT) and minor allele heterozygotes and homozygotes (CT/CC) in MESA samples.

Table 2 shows the SNP main effect and interaction *P*-values, beta, SNP minor allele frequencies (MAF), and sample sizes of race and sex-stratified *EBF1* GxE interactions with hip circumference in the MESA data set. Figure 1 shows the direction of race and sex-stratified interaction associations, which is consistent with the direction of association that we observed in our initial finding^10^ and its replications^11^, i.e., mean hip circumference increased with the increase in chronic psychosocial stress score for the minor allele group. The sex-stratified analysis showed that all observed significant *EBF1* gene-by-stress interactions were contributed by only female participants, it was significant only in Whites, and the average age of study samples was about 62 years (Table 2 and Fig. 1). Based on these results, our initial observation was that using a five-component stress measure, we might identify the *EBF1* gene-by-stress interaction only in White females that are relatively old. Not observing a significant interaction in Blacks in MESA study samples might be due to our inability to capture the adequate stress information through a MESA-like five-component stress measure.

**Table 2:** The observations of race and sex-stratified *EBF1* GxE (i.e., gene-by-stress or SNPxSTRESS) interactions with a hip circumference in the MESA data set.

Data set	Race	Sex	Mean age	*N*	Minor allele freq.	SNP beta	SNP *P-*value	GxE beta	GxE *P-*value
MESA	White	Both	62.68	2394	0.07	1.37	0.023	2.98	7.14E−09
MESA	White	Male	62.83	1153	0.06	0.24	0.740	0.34	0.624
MESA	White	Female	62.55	1241	0.07	2.38	0.012	4.42	7.97E−09
MESA	Black	Both	62.34	1575	0.02	−0.67	0.655	0.59	0.624
MESA	Black	Male	62.49	734	0.02	−2.20	0.286	0.59	0.764
MESA	Black	Female	62.21	841	0.02	0.88	0.680	−0.06	0.969

GxE *P*-value is SNPxSTRESS interaction *P*-value, and SNP *P*-value is SNP main-effect *P*-value with no stress or interaction term in the model.

### Hypothesis test 1

Table 3 shows the *P*-values of SNPxSTRESS term in JHS male, female, and both sexes combined for the self-rated eight-component stress summary score, a partial summary score using MESA-like items (i.e., job, relationship, caring for, and medical problem), and for the additional stress components (i.e., “racism/discrimination”, “living in neighborhood”, and “legal problems”) or an existing component but evaluated differently (i.e., not meeting basic needs). As hypothesized, the *EBF1* gene-by-stress interaction association in JHS females was significant for full stress summary score (*P* = 0.022), for the additional stress component “racism/discrimination” (*P* = 0.036), and moderately significant for differently evaluated financial strains component “not meeting basic needs” (*P* = 0.053). However, the association was not significant for the partial summary score using MESA-like components, i.e., making it equivalent to MESA-like self-rated or our synthetic stress measure, clearly indicating why we would not observe *EBF1* gene-by-stress interaction using a MESA-like self-rated or our synthetic stress measures in Black populations in other data sets.

**Table 3:** *EBF1* gene-by-stress (GxE)association with waist circumference in JHS.

Variable description	GxE	GxE	GxE	GxE	GxE	GxE
	Beta	*P*-val	Beta	*P*-val	Beta	*P*-val
	(All)	(All)	(Male)	(Male)	(Female)	(Female)
All (8) item summary score	1.67	0.013	2.45	0.12	1.72	0.022
MESA-alike 4-items summary score job, relationship, caring for, a medical problem	1.71	0.105	2.54	0.354	1.96	0.093
MESA-unlike 4-items summary score	2.96	0.014	5.06	0.067	2.57	0.052
Stress living in neighborhood	3.68	0.339	−13.16	0.327	6.39	0.123
Stress-related to legal problems	4.66	0.167	11.25	0.095	2.64	0.5
Stress from racism/discrimination	5.99	0.067	3.53	0.432	10.71	0.036
Stress meeting basic needs	6.68	0.016	8.01	0.104	6.48	0.053

### Hypothesis test 2

**Fig. 2:**
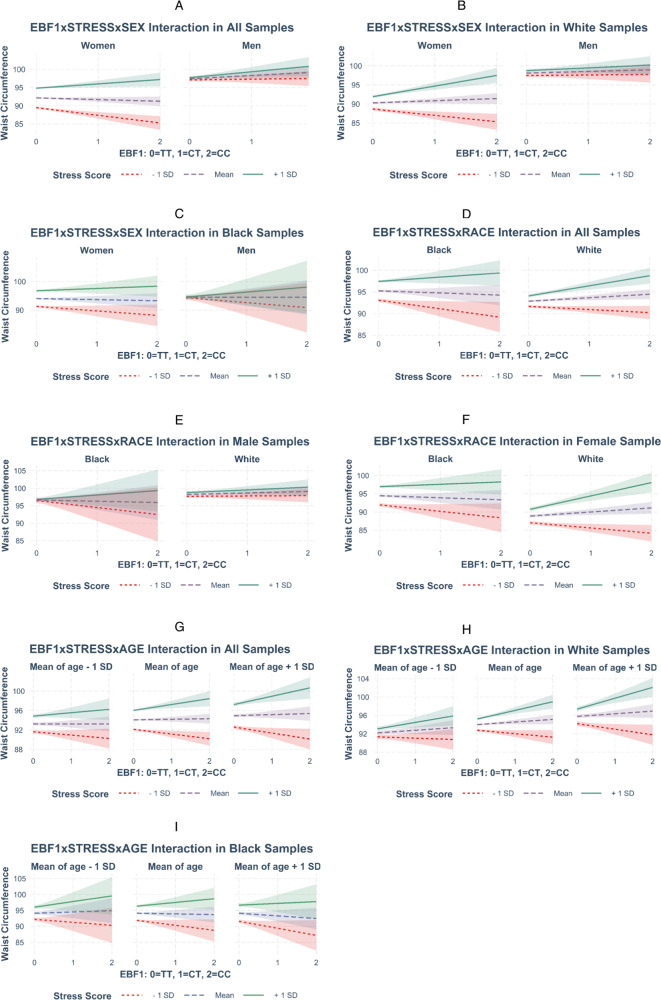
Johnson-Neyman interval plots for 3-way interaction. Each plot displays the estimated slope of *EBF1* genotype term predicting waist circumference when standardized stress score is its mean-1 SD, mean, and mean +1SD, given the third moderator sex, race, or age for each 3-way interactions listed in Table 4.

Table 4 shows the *P*-values for the associations of 3-way interaction terms with waist circumference using harmonized data sets, i.e., combined samples from ten studies in a mega-analysis. As hypothesized, the *P*-value (1.01e−03) for the association of 3-way interaction term SNPxSTRESSxSEX with waist circumference in White samples was statistically significant. However, the *P*-values for other 3-way interactions, i.e., SNPxSTRESSxRACE and SNPxSTRESSxAGE, were not statistically significant. We observed likely tentative associations for the 3-way interaction terms SNPxSTRESSxRACE and SNPxSTRESSxAGE in all-female samples (*P* = 0.103) and all White samples (*P* = 0.093), respectively. Figure 2 displays the 3-way interactions from Table 4 with Johnson–Neyman confidence bands. Each plot displays the estimated slope of *EBF1* genotype term predicting waist circumference when standardized stress score is low (mean-1 SD), medium (mean), and high (mean +1 SD), given sex, race, or age as the third moderator. The plots display distinctive estimated slopes of the *EBF1* genotype term, particularly for the 3-way interactions that showed statistically significant or tentative associations (Fig. 2a, b, f, h). These plots suggest that although the 3-way interaction tests with race and age did not achieve conventional levels of statistical significance, there may be some value in further pursuing these observed differences in additional samples. The *P*-values for the associations of 2-way interaction term SNPxSTRESS are shown in Supplementary Table S1. The SNP rs4704963 itself was not correlated with waist circumference (partial correlation coefficients = −0.0048─0.0149) in any stratum of the data set (Supplementary Table S1). In addition, stress was also not correlated with waist circumference for male participants in both Black and White samples (coefficients = 0.0231, 0.0509, respectively), but, as expected, it was moderately correlated for female participants in both ancestries (coefficients = 0.182, 0.137, respectively; Supplementary Table S1).

**Table 4:** 3-Way interactions: *EBF1* SNPXSTRESSXSEX, SNPXSTRESSXRACE, and SNPXSTRESSXAGE interaction association on WAIST CIRCUMFERENCE in harmonized data sets.

Race	Sex	Interaction term	*N*	Beta	*P*-value
White, Black	Male, female	SNPxSTRESSxSEX	28,026	−0.823	0.166
White	Male, female	SNPxSTRESSxSEX	15,027	−2.133	1.01e−03
Black	Male, female	SNPxSTRESSxSEX	12,999	0.514	0.768
White, Black	Male, female	SNPxSTRESSxRACE	28,026	0.052	0.937
White, Black	Male	SNPxSTRESSxRACE	9759	−1.364	0.323
White, Black	Female	SNPxSTRESSxRACE	18,267	1.316	0.103
White, Black	Male, female	SNPxSTRESSxAGE	28,026	0.029	0.217
White	Male, female	SNPxSTRESSxAGE	15,027	0.042	0.093
Black	Male, female	SNPxSTRESSxAGE	12,999	0.001	0.993

### Hypothesis test 3

**Fig. 3:**
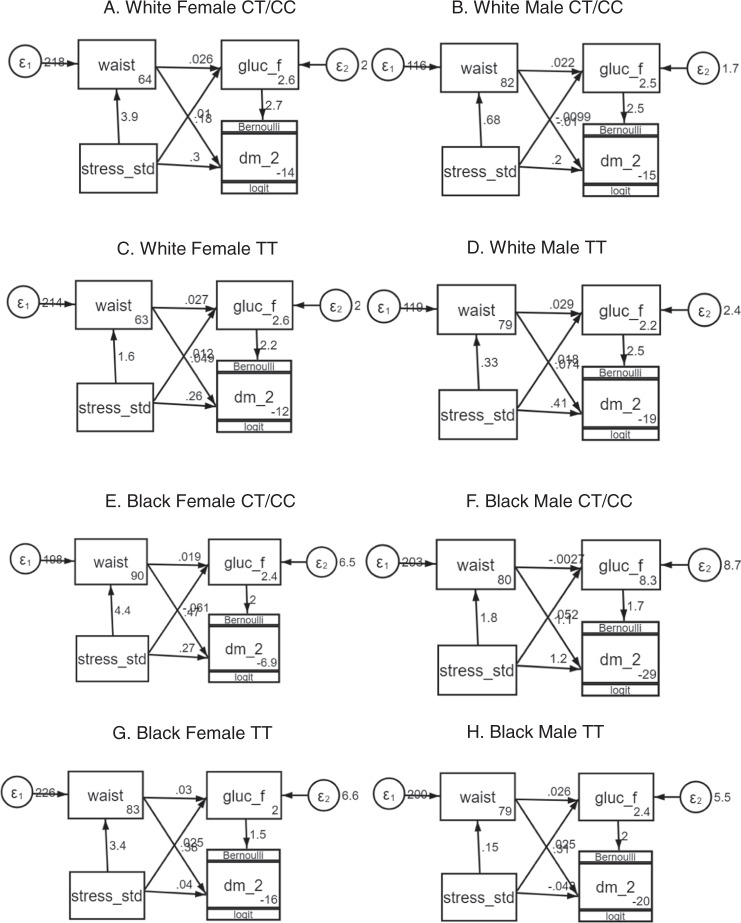
Structural equation model (SEM) path diagrams. The coefficients in SEMs stratified by race, sex, and *EBF1* rs4704963 genotypes, i.e., homozygote major allele (TT) and minor allele heterozygotes and homozygotes (CT/CC). The numbers shown at the side of each arrow and inside each rectangle are regression coefficient (slope) and intercept, respectively. The variables in the path diagram are stress_std: standardize stress measure, waist: waist circumference, gluc_f: fasting glucose, and dm_2: type 2 diabetes mellitus.

Table 5 shows the unstandardized coefficients and *P*-values of paths stress ⇒ waist, waist ⇒ glucose, glucose ⇒ DM, and indirect path stress ⇒ waist ⇒ glucose ⇒ DM for the race, sex, and *EBF1* SNP genotype-stratified analysis. Path coefficients can be interpreted as the expected change in the dependent (endogenous) variable for each one-unit change in the independent (exogenous) variable, similar to regression slope coefficients. We observed in general larger and significant paths for female carriers of *EBF1* minor allele heterozygotes and homozygotes (CT/CC) genotype compared to the carriers of major allele homozygotes (TT). The biggest difference between major and minor allele groups was clearly in the stress ⇒ waist path, all in the expected direction and consistent with our original finding^10^. For an example, a one-point increase in stress in the White female CT/CC group was associated with a 3.95 cm increase in waist circumference as compared to of 1.57 cm increase in the White female TT group, 0.68 cm increase in White male CT/CC group, and 0.33 cm in White male TT group. Figure 3 shows a graphical representation of complete path analysis in a different race, sex, and rs4704963 genotype groups. The indirect path stress ⇒ waist ⇒ glucose ⇒ DM was statistically significant and substantially larger in the White female CT/CC group (coefficient = 0.28, *P* = 0.009, 95% CI = 0.07–0.49) compared with the same path in the White female TT group (coefficient = 0.09, *P* = 0.03, 95% CI = 0.01–0.18). We also observed a similar pattern, i.e., larger coefficients of paths for Black female CT/CC group, however, the indirect path stress ⇒ waist ⇒ glucose ⇒ DM was not statistically significant (*P* = 0.089) for these samples (Table 5 and Fig. 3).

**Table 5:** Coefficients and *P*-values of paths in structural equation models (SEMs) stratified by race, sex, and *EBF1* rs4704963 genotypes, i.e., homozygote major allele (TT) and minor allele heterozygotes and homozygotes (CT/CC).

Race/sex	Genotype	Stress –> Waist		Waist –> GLUC		GLUC –> DM2		Indirect path (stress –> Waist –> GLUC –> DM2)	
		Coef	*P*-value	Coef	*P*-value	Coef	*P*-value	Coef	*P*-value
White female	CT/CC	3.947	<0.0001	0.026	<0.0001	2.680	<0.0001	0.280	0.009
	TT	1.571	<0.0001	0.027	<0.0001	2.188	<0.0001	0.093	0.028
White male	CT/CC	0.680	0.012	0.022	<0.0001	2.538	0.002	0.038	0.118
	TT	0.328	0.035	0.029	<0.0001	2.455	<0.0001	0.024	0.059
Black female	CT/CC	4.433	<0.0001	0.019	0.013	2.016	<0.0001	0.173	0.089
	TT	3.354	<0.0001	0.030	<0.0001	1.549	<0.0001	0.158	0.035
Black male	CT/CC	1.766	0.082	−0.003	0.878	1.745	0.003	−0.008	0.888
	TT	0.149	0.734	0.026	<0.0001	1.989	<0.0001	0.008	0.716

## Discussion

In our prior work^10^, we identified an *EBF1* gene-by-stress interaction associated with cardiometabolic risk factors (e.g., central obesity) in MESA White and Framingham Offspring data sets. Subsequently, we replicated this gene-by-stress interaction in three additional data sets using a synthetic stress measure that we created employing our algorithm based on the proxy indicators of MESA-like stress items^11^. Later, we performed data harmonization for chronic psychosocial stress, *EBF1* SNP rs4704963, and CVD-risk variables, including adiposity and hyperglycemia, in the ten studies that we used in the current work^8^. The data harmonization involved the construction of a synthetic stress measure^11^ in eight out of the ten studies that did not have a self-rated formal stress measure, while two studies (MESA, JHS) had a formal self-rated stress measure^8^. As presented in our previous work^8^, the broad domains of psychosocial stress that we have used were consistent with that of others using similar stress measures -- including measures explicitly designed to assess stress^12,24^—and were apparently sufficient to capture life stress, even when not all domains were present in the synthetic measure^11^.

In the present study, we replicated the *EBF1* gene-by-stress interaction in JHS Black samples (*P* = 0.022), in addition to previously observed significant associations in White samples^10,11^. The replication of the *EBF1* gene-by-stress interaction in Black females in JHS was apparently due to the additional components of “racism/discrimination” and “not meeting basic needs” (*P*s = 0.036, 0.053, respectively), which were unique to the JHS stress assessment. This may be due to the added relevance of these additional stress components in Black populations. However, the JHS observations may be study-specific that may not be generalized until more work elucidates these interactions in Black samples. We did not have all additional JHS indicators in our synthetic stress, therefore, it was not possible for us to test the hypothesis 1 in the harmonized data set^8^, combining all samples from multiple studies.

The formal testing of 3-way interactions involving SNP, stress, and sex, race, and age on waist circumference resulted in a statistically significant association only for SNPxSTRESSxSEX term (*P* = 1.01e−03) in White samples. The *P*-values for 3-way interaction terms SNPxSTRESSxRACE and SNPxSTRESSxAGE did not reach conventional statistical significance level in any of the stratified categories. Detecting statistically significant interactions are known to be challenging, depending on several factors, including sample size, the distribution of the interaction component variables (e.g., minor allele or genotype frequency or in the case of continuous variables, the thinness of the tails), discovery power, phenotypic information contents, and related differential biological mechanism(s) in specific sample groups. The information regarding these factors with respect to an observed or unobserved association may elucidate the precision of an interaction. In the *EBF1* gene-by-stress interaction case, the minor allele frequency in White samples was more than three times larger than that was for Black samples. Replication in JHS also revealed that population specificity of stress measures might make a difference, despite the low sample size. Any observation of 3-way interactions clearly depended on the differential association of the SNPxSTRESS term over the third term, i.e., sex, race, or age. While sex and race have well-defined categories (i.e., male/female, white/black) to reveal which population group(s) contribute to the SNPxSTRESS term association (e.g., white females, Supplementary Table S1), it is generally not recommended to dichotomize continuous variable^26^. We, therefore, used age as a continuous variable in our analyses (Tables 2–5) and observed only a tentative 3-way SNPxSTRESSxAGE interaction association in White samples (*P* = 0.093). More studies are needed to evaluate the differential relationship of age over the gene-by-stress interaction and its influence on the development of cardiometabolic risks.

Structural equation path analysis for possible mediated causal paths from stress to obesity to fasting blood glucose to type 2 diabetes mellitus, a cardiometabolic disease risk factor, revealed that the direct path stress ⇒ waist was substantially larger and significant in White and Black female CT/CC groups as compared to other stratified groups. Also, the indirect path stress ⇒ waist ⇒ glucose ⇒ DM was largest and statistically significant in the White female CT/CC group. Our inability to observe a similar statistically significant indirect path in the Black female CT/CC group might be due in parts to the lack of a population-specific stress measure component (e.g., racism or discrimination) in the harmonized data set and/or low minor allele frequency of the *EBF1* SNP in Black samples.

The use of the harmonized data set from all studies helped us develop robust and stable estimates of the interactions among an individual’s genetic makeup for *EBF1* SNP rs4704963 and complex physiological aspects of sex, race, and age. The *EBF1*xSTRESS term *P*-value (4.68E−06) for White samples from the multi-study large harmonized data set (Supplementary Table S1) was not as strong as the *P*-value of the same term (7.14E−09) for the relatively much smaller MESA data set in our discovery GWAS analysis^10^. This indicated that the analysis using a larger sample size may result in a stable and more generalizable association, not necessarily a stronger association in terms of *P*-value.

In conclusion, the use of synthetic stress measures in the harmonized data set has shown that if a self-rated chronic psychosocial stress score was not obtained at the time of initial sample collection, we could still use available data to compute a synthetic stress score retrospectively. Our work provides additional confirmation, including in Black samples, that common variation in *EBF1* may contribute to inter-individual differences in human obesity in the presence of stress, and that the gene-by-stress interaction differs depending on the sex of participants in both White and Black samples. The observed associations appeared to be present only among female participants. There also was preliminary evidence that at least some of the associations may vary across the lifespan, but more work is needed for confirmation. A MESA-like 5-component stress measure does not appear to capture the key life-stress indicator in the Black population, in which a measure of discrimination appears necessary. We observed a substantially larger and significant direct path stress ⇒ waist in White and Black female *EBF1* minor allele carriers as compared to other stratified groups and also the largest and significant indirect path stress ⇒ waist ⇒ glucose ⇒ DM in the White female *EBF1* minor allele group. Our work may provide a foundation to the precision medicine framework related to *EBF1* gene-by-stress interactions, which in turn may lead to therapeutic intervention focused on a precise risk group, i.e., only female, mostly Whites, and possibly older individuals.

## Supplementary information

Supplemental Material

## Data Availability

The public-access data sets used in this study can be obtained from the dbGaP/database of Genotypes and Phenotypes/National Center for Biotechnology Information, National Library of Medicine (NCBI/NLM)/https://www.ncbi.nlm.nih.gov/gap through authorized access approved by NIH Data Access Committee. The Duke data sets, however, are available for collaborative use upon reasonable request of collaborations with authors on the permission of the respective Study Committee and Duke IRB.
