# Supplementary material for: Evaluating the precision of EBF1 SNP x stress interaction association: sex, race, and age differences in a big harmonized data set of 28,026 participants
Source: Transl Psychiatry. 2020 Oct 20;10:351. doi: 10.1038/s41398-020-01028-5 (PMC7572375; doi:10.1038/s41398-020-01028-5)
Supplement: Supplementary file 1 — Supplemental Material [file 41398_2020_1028_MOESM1_ESM.pdf]

Evaluating the precision of *EBF1* SNP x Stress Interaction Association: Sex, Race, and Age Differences in a Big Harmonized Dataset of 28,026 participants

Abanish Singh, Ph.D.*[,1,2], Michael A. Babyak, Ph.D.[1,2], Mario Sims, Ph.D.[3], Solomon K. Musani, Ph.D.[3], Beverly H. Brummett, Ph.D.[1,2], Rong Jiang, Ph.D.[1,2], William E. Kraus, M.D.[4,5], Svati H. Shah, M.D.[4,5], Ilene C. Siegler, Ph.D. [1,2], Elizabeth R. Hauser, Ph.D. [4,6], Redford B. Williams, M.D.[1,2]

(1) Behavioral Medicine Research Center, Duke University School of Medicine, Durham, NC, (2) Department of Psychiatry and Behavioral Sciences, Duke University School of Medicine, Durham, NC, (3) Department of Medicine of the University of Mississippi Medical Center, Jackson, MS, (4) Duke Molecular Physiology Institute, Duke University School of Medicine, Durham, NC, (5) Department of Medicine, Duke University School of Medicine, Durham, NC, (6) Department of Biostatistics and Bioinformatics, Duke University School of Medicine, Durham, NC

*Corresponding Author:

Abanish Singh, Ph.D.

Duke University School of Medicine

Duke Box 104775, Durham, NC 27701

Email: abanish.singh@duke.edu

SUPPLEMENTAL MATERIALS

Study Populations:

Jackson Heart Study (JHS): The JHS is a large, community-based, observational study that was designed to explore reasons for the prevalence of cardiovascular disease among African Americans (Sempos et al., 1999). The study participants were recruited from urban and rural areas of the Jackson MS, metropolitan statistical area (MSA). The study aimed to identify genetic factors that affect cardiometabolic risk factors in African Americans.

The Women's Health Initiative (WHI): WHI is a long-term national health study dedicated to developing prevention strategies for heart disease, breast and colorectal cancer, and osteoporotic fractures in postmenopausal women (The WHI Study Group, 1998) . The groundbreaking results from this study have made significant contributions towards the care and prevention of some of the major health conditions affecting postmenopausal women.

The Coronary Artery Risk Development in Young Adults Study (CARDIA): CARDIA was designed to study the etiology and natural history of cardiovascular disease beginning in young adulthood (Friedman et al., 1988). The CARDIA study participants' selection constituted approximately the same number of people in subgroups of race, gender, education.

Atherosclerosis Risk in Communities Study (ARIC): ARIC is a prospective epidemiologic study focused to investigate the etiology and natural history of atherosclerosis and demographic variation in cardiovascular risk factors, medical care, and disease(The ARIC Investigators, 1989). The study examined atherosclerosis by direct observation and by use of modern biochemistry. The components of the study included identification, investigation, and diagnosis of clinical events through home interviews, clinic examinations, and annual telephone follow-ups.

Framingham Offspring Cohort: We used the Generation 2 (or Offspring) dataset from the Framingham Heart Study Cohort for this work (Feinleib et al., 1975) because of availability of psychosocial measurements and genetic data. The second-generation cohort included adult children (and their spouses) of the original participants. The cohort is primarily White.

Multi-Ethnic Study of Atherosclerosis (MESA): MESA was designed to study the CVD risk factors that predict progression of the clinically observable or subclinical cardiovascular disease (Bild et al., 2002). The dataset included a well-characterized self-rated chronic psychosocial stress summary measure "chronic burden", quantified on an ordinal scale of 0 to 5 (0, 1, 2, 3, 4, and 5) based on questionnaires in five domains including questions about ongoing serious health problems, serious health problems with someone close, work-related problems, financial strains, and difficulties in relationships (Shivpuri et al., 2012; Singh et al., 2015a; Singh et al., 2015b).

Studies of a Targeted Risk Reduction Intervention through Defined Exercise (STRRIDE): The Duke STRRIDE cohort includes two studies: STRRIDE – Aerobic Training / Resistance Training (AT/RT) and STRRIDE pre-diabetes (PD). STRRIDE AT/RT study was designed to compare the effects of aerobic training (AT) and resistance training (RT) and the full combination (AT/RT) on central ectopic fat and liver enzymes and fasting insulin resistance by homeostatic model assessment (HOMA) (Slentz et al., 2011). The purpose of the STRRIDE-PD study was to compare the effects of different amounts and intensities of exercise training programs without diet to an exercise and diet program modeled after the first six months of the Diabetes Prevention Program (DPP) (Slentz et al., 2016).

Duke Caregiver Study (DCS): This study was conducted at the Duke University Medical Center that included data from family caregivers of a relative with Alzheimer's disease or other dementia and non-caregiving control (Siegler et al., 2010).

Duke Family Heart Study (DFHS): This study was conducted at Duke University Medical Center under the approval of Duke IRB to study the effect of genetic variation on the relationship between psychosocial and cardiovascular risk factors (Brummett et al., 2010).

Proxy item of Synthetic Chronic Psychosocial Stress: For CARDIA we identified the proxy items related to diseases or health problems, financial success, marital status, job problems (fired, demoted, and laid off). In ARIC, we used income, health compare to other people of same age, and marital status. In STRRIDE, we identified proxy items related to general health and job accomplishment (i.e., job demand). In Duke Caregiver Study, we used proxy items household income; spouse related hassles; hassles related to health or well-being of a family member; self-health related hassles; and a score of hassles related to job. In DFHS, we used total income and a score for work difficulties (job insecurity, lack of career prospects, issues with support at work, and job dissatisfaction). In Framingham Offspring Cohort, we identified total family income; job insecurity and physiological job demand scale; marital disagreement; and spouse's heart attack, stroke, and heart disease-related death.

Structural Equation Path Modelling:

In structural equation modellings (SEM), we analyze structural relationships through multivariate analysis. The analysis involves mainly two type of variables, i.e., endogenous and exogenous variables, which are equivalent dependent and independent variables, respectively. The SEM adds another type of variable associated to an endogenous variable, the error variable, which are latent exogenous variables with a fixed-unit path coefficient and are denoted with an e. prefix. In the analysis, we hypothesize a path for a possible mediated causal association, for example that the association between x and y is mediated by the variable m and we estimate the mediated or indirect effect of x on y by the multiple simultaneous regressions y = b1*x +

b2*m and m = b3*x, where b1, b2, and b3 represent regression slopes. The paths x → m → y with the direct effects can be represented graphically using directed arrows and the regression slope between each pair of variables. The magnitude of a mediating effect is then calculated by taking the product of all path coefficients along a given proposed path. In the example above, the indirect effect (i.e., x → m → y) would be the product of b2 and b3. The standard error for the product is then used to test the null hypothesis that the product is zero.

Power Analysis:

Applying the effect sizes observed in the initial discovery of *EBF1* GxE association with hip circumference (Singh et al., 2015b) at Bonferroni correction significance level (MAF=0.07, SD_e=1, β_g=-2, β_e=4.3, β_{ge}=3, $\alpha=5x10^{-8}$) the sample size needed is 3151 to achieve 80% power (Quanto software, http://biostats.usc.edu/Quanto.html). Thus, our harmonized dataset of 28,026 participants offers adequate statistical power to detect the GxE interaction association.

Acknowledgments:

The JHS is supported and conducted in collaboration with Jackson State University (HHSN268201800013I), Tougaloo College (HHSN268201800014I), the Mississippi State Department of Health (HHSN268201800015I) and the University of Mississippi Medical Center (HHSN268201800010I, HHSN268201800011I and HHSN268201800012I) contracts from the National Heart, Lung, and Blood Institute (NHLBI) and the National Institute for Minority Health and Health Disparities (NIMHD).

The WHI program is funded by the National Heart, Lung, and Blood Institute, National Institutes of Health, U.S. Department of Health and Human Services through contracts N01WH22110, 24152, 32100-2, 32105-6, 32108-9, 32111-13, 32115, 32118-32119,

32122, 42107-26, 42129-32, and 44221. This manuscript was not prepared in collaboration with investigators of the WHI and does not necessarily reflect the opinions of the WHI investigators or the NHLBI.

The CARDIA is conducted and supported by the National Heart, Lung, and Blood Institute (NHLBI) in collaboration with the University of Alabama at Birmingham (N01-HC95095 & N01-HC48047), University of Minnesota (N01-HC48048), Northwestern University (N01-HC48049), and Kaiser Foundation Research Institute (N01-HC48050). This manuscript was not approved by CARDIA. The opinions and conclusions contained in this publication are solely those of the authors, and are not endorsed by CARDIA or the NHLBI and should not be assumed to reflect the opinions or conclusions of either.

The ARIC has been funded in whole or in part with Federal funds from the National Heart, Lung, and Blood Institute, National Institute of Health, Department of Health and Human Services, under contract numbers (HHSN268201700001I, HHSN268201700002I, HHSN268201700003I, HHSN268201700004I, and HHSN268201700005I). This manuscript was not prepared in collaboration with investigators of the ARIC and does not necessarily reflect the opinions of the ARIC investigators or the NHLBI.

The Framingham Heart Study is conducted and supported by the National Heart, Lung, and Blood Institute (NHLBI) in collaboration with Boston University (Contract No. N01-HC-25195, HHSN268201500001I and 75N92019D00031). This manuscript was not prepared in collaboration with investigators of the Framingham Heart Study and does not necessarily reflect the opinions or views of the Framingham Heart Study, Boston University, or NHLBI.

MESA and the MESA SHARe project are conducted and supported by the National Heart, Lung, and Blood Institute (NHLBI) in collaboration with MESA investigators. Support for MESA is provided by contracts N01-HC95159, N01-HC-95160, N01-HC-95161, N01-HC-95162, N01-HC-95163, N01-HC-95164, N01-HC-95165, N01-HC95166, N01-HC-95167, N01-HC-95168, N01-HC-95169 and CTSA UL1-RR-024156. This manuscript was not prepared in collaboration with investigators of the MESA and does not necessarily reflect the opinions of the MESA investigators or the NHLBI. This STRRIDE AT/RT was funded by National Heart, Lung, and Blood Institute Grant 2R01-HL-057354 (clinical trial registration no. NCT00275145) and the STRRIDE-PD study was funded by National Institutes for Health National Institute of Diabetes and Digestive and Kidney Diseases grant R01DK081559.

The DCS study was supported by the Alzheimer's Association (IIRG-08-89565), the National Institute on Aging with co-funding by the National Institute of Environmental Health Sciences, the National Institute of Mental Health (R01 AG19605), the National Heart Lung and Blood Institute (P01HL36587), The Claude D. Pepper Older Americans Independence Center (P30 AG028716), and the Duke Behavioral Medicine Research Center.

The DFHS study was supported by the by National Heart, Lung, and Blood Institutes grant 3P01 HL036587, the Clinical Research Unit grant M01RR30l, and by the Duke Behavioral Medicine Research Center.

TABLE S1: A follow-up of significant 3-way interactions from Table 4: Sex, race, and age stratified SNPxSTRESS association with waist circumference in combined harmonized datasets and the coefficients of model variables-adjusted partial correlation of SNP and stress with waist circumference.

Race	Sex	Partial correlation coefficients		2-Way Interaction Analysis			
		SNP	Stress	Interaction term	N	Beta	*P*-value
WHITE	MALE, FEMALE	0.0134	0.1078	SNPxSTRESS	15,027	1.473	**4.68E-06**
WHITE	MALE	0.0132	0.0509	SNPxSTRESS	7,056	0.343	0.392
WHITE	FEMALE	0.0149	0.137	SNPxSTRESS	7,971	2.430	**5.09E-07**
BLACK	MALE, FEMALE	-0.0037	0.153	SNPxSTRESS	12,999	1.330	**0.025**
BLACK	MALE	0.0014	0.0231	SNPxSTRESS	2,703	1.715	0.267
BLACK	FEMALE	-0.0048	0.182	SNPxSTRESS	10,296	1.127	0.08

REFERENCES:

Bild, D. E., et al. (2002). Multi-Ethnic Study of Atherosclerosis: Objectives and Design. *American Journal of Epidemiology, 156*(9), 871-881.

Brummett, B. H., et al. (2010). Associations of depressive symptoms, trait hostility, and gender with C-reactive protein and interleukin-6 response following emotion recall. *Psychosomatic Medicine, 72*(4), 333-339.

Feinleib, M., Kannel, W. B., Garrison, R. J., McNamara, P. M., & Castelli, W. P. (1975). The framingham offspring study. Design and preliminary data. *Preventive Medicine, 4*(4), 518-525.

Friedman, G. D., et al. (1988). Cardia: study design, recruitment, and some characteristics of the examined subjects. *Journal of Clinical Epidemiology, 41*(11), 1105-1116.

Sempos, C. T., Bild, D. E., & Manolio, T. A. (1999). Overview of the Jackson Heart Study: A Study of Cardiovascular Diseases in African American Men and Women. *The American Journal of the Medical Sciences, 317*(3), 142-146.

Shivpuri, S., Gallo, L. C., Crouse, J. R., & Allison, M. A. (2012). The Association Between Chronic Stress Type and C-Reactive Protein in the Multi-Ethnic Study of Atherosclerosis (MESA): Does Gender Make a Difference? *Journal of Behavioral Medicine, 35*(1), 74-85.

Siegler, I. C., Brummett, B. H., Williams, R. B., Haney, T. L., & Dilworth-Anderson, P. (2010). Caregiving, residence, race, and depressive symptoms. *Aging & mental health, 14*(7), 771-778.

Singh, A., et al. (2015a). Computing a Synthetic Chronic Psychosocial Stress Measurement in Multiple Datasets and its Application in the Replication of G × E Interactions of the EBF1 Gene. *Genetic Epidemiology, 39*(6), 489-497.

Singh, A., et al. (2015b). Gene by stress genome-wide interaction analysis and path analysis identify EBF1 as a cardiovascular and metabolic risk gene. *Eur J Hum Genet, 23*(6), 854-862.

Slentz, C. A., et al. (2016). Effects of exercise training alone vs a combined exercise and nutritional lifestyle intervention on glucose homeostasis in prediabetic individuals: a randomised controlled trial. *Diabetologia, 59*(10), 2088-2098.

Slentz, C. A., et al. (2011). Effects of aerobic vs. resistance training on visceral and liver fat stores, liver enzymes, and insulin resistance by HOMA in overweight adults from STRRIDE AT/RT. *American Journal of Physiology - Endocrinology And Metabolism, 301*(5), E1033.

The ARIC Investigators. (1989). THE ATHEROSCLEROSIS RISK IN COMMUNIT (ARIC) STUI)Y: DESIGN AND OBJECTWES. *American Journal of Epidemiology, 129*(4), 687-702.

The WHI Study Group. (1998). Design of the Women's Health Initiative Clinical Trial and Observational Study. *Controlled Clinical Trials, 19*(1), 61-109.
